# Energy metabolism dysregulation, cerebrovascular aging, and time-restricted eating: Current evidence and proof-of-concept findings

**DOI:** 10.1093/pnasnexus/pgae505

**Published:** 2024-11-08

**Authors:** Ana Clara da C Pinaffi-Langley, Camila B Pinto, Peter Mukli, Anna Peterfi, Zalan Kaposzta, Cameron D Owens, Zsofia Szarvas, Mihaly Muranyi, Cheryl Adams, Ali Shahriari, Priya Balasubramanian, Zoltan Ungvari, Anna Csiszar, Shannon Conley, Norman G Hord, Leah Anderson, Stefano Tarantini, Andriy Yabluchanskiy

**Affiliations:** Oklahoma Center for Geroscience and Healthy Brain Aging, University of Oklahoma Health Sciences, Oklahoma City, OK 73104, USA; Department of Nutritional Sciences, College of Allied Health, University of Oklahoma Health Sciences, Oklahoma City, OK 73104, USA; Oklahoma Center for Geroscience and Healthy Brain Aging, University of Oklahoma Health Sciences, Oklahoma City, OK 73104, USA; Department of Neurosurgery, Vascular Cognitive Impairment and Neurodegeneration Program, University of Oklahoma Health Sciences, Oklahoma City, OK 73104, USA; Oklahoma Center for Geroscience and Healthy Brain Aging, University of Oklahoma Health Sciences, Oklahoma City, OK 73104, USA; Department of Neurosurgery, Vascular Cognitive Impairment and Neurodegeneration Program, University of Oklahoma Health Sciences, Oklahoma City, OK 73104, USA; Department of Public Health, International Training Program in Geroscience, Doctoral School of Basic and Translational Medicine, Semmelweis University, Budapest H-1085, Hungary; Department of Physiology, Faculty of Medicine, Semmelweis University, Budapest H-1085, Hungary; Oklahoma Center for Geroscience and Healthy Brain Aging, University of Oklahoma Health Sciences, Oklahoma City, OK 73104, USA; Department of Neurosurgery, Vascular Cognitive Impairment and Neurodegeneration Program, University of Oklahoma Health Sciences, Oklahoma City, OK 73104, USA; Department of Public Health, International Training Program in Geroscience, Doctoral School of Basic and Translational Medicine, Semmelweis University, Budapest H-1085, Hungary; Oklahoma Center for Geroscience and Healthy Brain Aging, University of Oklahoma Health Sciences, Oklahoma City, OK 73104, USA; Department of Neurosurgery, Vascular Cognitive Impairment and Neurodegeneration Program, University of Oklahoma Health Sciences, Oklahoma City, OK 73104, USA; Department of Public Health, International Training Program in Geroscience, Doctoral School of Basic and Translational Medicine, Semmelweis University, Budapest H-1085, Hungary; Oklahoma Center for Geroscience and Healthy Brain Aging, University of Oklahoma Health Sciences, Oklahoma City, OK 73104, USA; Department of Neurosurgery, Vascular Cognitive Impairment and Neurodegeneration Program, University of Oklahoma Health Sciences, Oklahoma City, OK 73104, USA; Oklahoma Center for Geroscience and Healthy Brain Aging, University of Oklahoma Health Sciences, Oklahoma City, OK 73104, USA; Department of Neurosurgery, Vascular Cognitive Impairment and Neurodegeneration Program, University of Oklahoma Health Sciences, Oklahoma City, OK 73104, USA; Department of Public Health, International Training Program in Geroscience, Doctoral School of Basic and Translational Medicine, Semmelweis University, Budapest H-1085, Hungary; Oklahoma Center for Geroscience and Healthy Brain Aging, University of Oklahoma Health Sciences, Oklahoma City, OK 73104, USA; Department of Neurosurgery, Vascular Cognitive Impairment and Neurodegeneration Program, University of Oklahoma Health Sciences, Oklahoma City, OK 73104, USA; Oklahoma Shared Clinical and Translational Resources, University of Oklahoma Health Sciences, Oklahoma City, OK 73104, USA; Oklahoma Shared Clinical and Translational Resources, University of Oklahoma Health Sciences, Oklahoma City, OK 73104, USA; Oklahoma Center for Geroscience and Healthy Brain Aging, University of Oklahoma Health Sciences, Oklahoma City, OK 73104, USA; Department of Neurosurgery, Vascular Cognitive Impairment and Neurodegeneration Program, University of Oklahoma Health Sciences, Oklahoma City, OK 73104, USA; Oklahoma Center for Geroscience and Healthy Brain Aging, University of Oklahoma Health Sciences, Oklahoma City, OK 73104, USA; Department of Neurosurgery, Vascular Cognitive Impairment and Neurodegeneration Program, University of Oklahoma Health Sciences, Oklahoma City, OK 73104, USA; Department of Public Health, International Training Program in Geroscience, Doctoral School of Basic and Translational Medicine, Semmelweis University, Budapest H-1085, Hungary; Department of Health Promotion Sciences, College of Public Health, University of Oklahoma Health Sciences, Oklahoma City, OK 73104, USA; Oklahoma Center for Geroscience and Healthy Brain Aging, University of Oklahoma Health Sciences, Oklahoma City, OK 73104, USA; Department of Neurosurgery, Vascular Cognitive Impairment and Neurodegeneration Program, University of Oklahoma Health Sciences, Oklahoma City, OK 73104, USA; Oklahoma Center for Geroscience and Healthy Brain Aging, University of Oklahoma Health Sciences, Oklahoma City, OK 73104, USA; Department of Cell Biology, College of Medicine, University of Oklahoma Health Sciences, Oklahoma City, OK 73104, USA; Department of Nutritional Sciences, College of Education and Human Sciences, Oklahoma State University, Stillwater, OK 74078, USA; Department of Nutritional Sciences, College of Allied Health, University of Oklahoma Health Sciences, Oklahoma City, OK 73104, USA; Oklahoma Center for Geroscience and Healthy Brain Aging, University of Oklahoma Health Sciences, Oklahoma City, OK 73104, USA; Department of Neurosurgery, Vascular Cognitive Impairment and Neurodegeneration Program, University of Oklahoma Health Sciences, Oklahoma City, OK 73104, USA; Department of Public Health, International Training Program in Geroscience, Doctoral School of Basic and Translational Medicine, Semmelweis University, Budapest H-1085, Hungary; Department of Health Promotion Sciences, College of Public Health, University of Oklahoma Health Sciences, Oklahoma City, OK 73104, USA; Peggy and Charles Stephenson Cancer Center, University of Oklahoma Health Sciences, Oklahoma City, OK 73104, USA; Oklahoma Center for Geroscience and Healthy Brain Aging, University of Oklahoma Health Sciences, Oklahoma City, OK 73104, USA; Department of Neurosurgery, Vascular Cognitive Impairment and Neurodegeneration Program, University of Oklahoma Health Sciences, Oklahoma City, OK 73104, USA; Department of Health Promotion Sciences, College of Public Health, University of Oklahoma Health Sciences, Oklahoma City, OK 73104, USA; Peggy and Charles Stephenson Cancer Center, University of Oklahoma Health Sciences, Oklahoma City, OK 73104, USA

**Keywords:** cerebrovasculature, dietary intervention, energy metabolism, intermittent fasting, older adults

## Abstract

Dysregulated energy metabolism is a hallmark of aging, including brain aging; thus, strategies to restore normal metabolic regulation are at the forefront of aging research. Intermittent fasting, particularly time-restricted eating (TRE), is one of these strategies. Despite its well-established effectiveness in improving metabolic outcomes in older adults, the effect of TRE on preserving or improving cerebrovascular health during aging remains underexplored. We explored how aging itself affects energy metabolism and contextualized these age-related changes to cerebrovascular health. We also conducted a literature search on PubMed and Scopus to identify and summarize current studies on TRE in older adults. Finally, we provided preliminary data from our proof-of-concept pilot trial on the effect of 6-month TRE on cerebrovascular health in older adults. Current evidence shows the potential of TRE to improve energy metabolism and physiological outcomes in older adults. TRE may improve cerebrovascular function indirectly due to its effect on glucose homeostasis. However, to date, direct evidence of the effect of TRE on cerebrovascular parameters is lacking. TRE is a well-tolerated and promising dietary intervention for promoting and maintaining cerebrovascular health in older adults. Further studies on TRE in older adults must be better controlled for energy balance to elucidate its independent effects from those of caloric restriction.

## Introduction

Dysregulated energy metabolism underlies and integrates several hallmarks of aging (1, 2). As such, strategies to reestablish a well-regulated metabolism are at the forefront of aging research. Chief among these strategies is intermittent fasting, which refers to the practice of restricting one's caloric intake for extended periods of time (from hours to days at a time). In particular, time-restricted eating (TRE) is emerging as a feasible and safe intermittent fasting modality for older adults (3, 4). In TRE, the day is broken down into periods of free eating (1–10 h) and periods of fasting (23–14 h), with most common regimens in the range of 14 to 16 h of fasting. The daily rhythmicity and regimen flexibility of TRE lend it special interest for implementation in clinical practice. Moreover, recent evidence indicates that TRE can improve cardiometabolic health outcomes without caloric restriction (3, 5). This is especially important for older adults because unintended weight loss and undernutrition increase the risk of frailty and mortality in this population (6–8).

Another priority of aging research lies in the maintenance of cognitive health during aging. This priority effort is propelled by the recent estimates that show a projected increase of 50% in the number of older adults living with dementia by 2040 (9). Despite the increasing interest, to date, few human studies have explored the effect of TRE on cerebrovascular and cognitive health in older adults. In an observational study, Currenti et al. (10) reported that older Italian adults who habitually maintained an eating pattern of 10 h or less between first and last meal were less likely to have cognitive impairment than their counterparts. In contrast, Li et al. (11) found that an eating period of 10 h or less was associated with worse cognitive performance in older Chinese adults. These contrasting results underscore the need for clinical investigations designed to elucidate the role of TRE in cerebrovascular health in older adults.

In this review, we aimed to explore how aging itself affects energy metabolism and to contextualize these age-related changes to cerebrovascular health. We also aimed to summarize current studies on TRE in older adults as well as to propose a framework for future studies examining the effect of TRE on cerebrovascular aging. Finally, we provided preliminary data from our proof-of-concept pilot trial on the effect of long-term TRE on cerebrovascular health in older adults. This review will inform clinicians and researchers on important considerations when designing dietary interventions for older adults, which has the potential to increase the translatability of such strategies for real-world applications.

## Energy metabolism: the fed-fasting cycle and aging

Resilience is an intrinsic property of complex systems reflecting its capacity of recovering from stressors (12). This property is aptly illustrated by the fed-fasting cycle, in which interconnected systemic pathways adapt to cycles of nutrient availability and scarcity to maintain glucose homeostasis as well as adequate protein and lipid turnover cycles. After a meal, the increase in blood glucose (along with other preabsorptive mechanisms such as cephalic responses and incretin signaling) signals pancreatic beta cells to release a bolus of insulin, an event that inaugurates the fed state and associated anabolic reactions. The main anabolic reactions directly affected by insulin are organ-dependent: (ⅰ) in the liver, insulin activates glucose storage mechanisms (glycogenesis) and up-regulates lipogenic gene expression, while down-regulating gluconeogenic gene expression; (ⅱ) in white adipose cells, insulin increases glucose uptake and utilization for energy and activates fat storage mechanisms (lipogenesis) while decreasing lipolysis; and (ⅲ) in skeletal muscle, insulin increases glucose uptake and utilization for energy and activates glycogenesis (13). On a cellular level, insulin signaling initiates both mitogenic and metabolic cascades. Relevant to energy metabolism, the metabolic cascade involving insulin receptor substrate–phosphoinositide-3-kinase (PI3K)–Akt is a key activator of the mammalian target of rapamycin complex 1 (mTORC1). mTORC1 is a master cellular nutrient sensor involved in the regulation of cell growth, energy metabolism, and protein and lipid synthesis (14).

As exogenous glucose is depleted (0–3 h after a meal), the decrease in glucose levels in the portal vein signals pancreatic alpha cells to release glucagon via a combination of metabolic and neuronal inputs (15), inaugurating the post-absorptive state and initiating catabolic reactions to maintain adequate blood glucose levels in the absence of exogenous fuel sources. The liver is the main target organ for glucagon's direct effects, where it stimulates glycogenolysis and gluconeogenesis to increase the glucose output of the liver while simultaneously inhibiting glycolysis and glycogenesis. At this stage, the main source of blood glucose is hepatic glycogenolysis. Glucagon also increases fatty acid beta-oxidation in the mitochondria and decreases lipid storage in hepatocytes. Indirectly, glucagon increases lipolysis in white adipose tissue and decreases glucose uptake in skeletal muscle. Glucagon signaling involves the adenylyl cyclase–cyclic adenosine monophosphate (cAMP)–protein kinase A cascade, which activates transcription factors related to gluconeogenesis and beta-oxidation. One of the ways through which adenylyl cyclase-mediated ATP conversion to cAMP can activate the energy sensing kinase, AMP-activated protein kinase (AMPK), is through an increase in the AMP-to-ATP ratio due to the degradation of cAMP by phosphodiesterases (16). Of note, cAMP-induced AMPK activation is one of several AMPK inputs (17), and its activation pattern can vary depending on tissue and, more broadly, on fitness level. For instance, AMPK is activated in skeletal muscle after as little as 12 h of fasting in trained humans (18). AMPK activation further stimulates fatty acid beta-oxidation and inhibits lipid synthesis from acetyl-CoA (19). Further, decreased glycolysis increases the NAD^+^ -to-NADH ratio, thereby triggering the activation of sirtuins. These histone deacetylases participate in glucose and lipid metabolism during fasting periods, improving mitochondrial activity, and decreasing glucose utilization while increasing lipid utilization in skeletal muscle and the liver (20). These metabolic changes preserve blood glucose for utilization by the brain, nerve cells, and red blood cells, while other tissues and cells (such as skeletal and cardiac muscle) that are well adapted to using fatty acids as energy sources decrease glucose uptake and utilization.

The post-absorptive state lasts between 3 and 18 h. As hepatic glycogen stores decrease, we enter the fasting state, where the main source of blood glucose is hepatic gluconeogenesis (hepatic glycogenolysis still contributes to blood glucose, but to a lesser degree) (21). The main source of gluconeogenic precursors during this stage is skeletal muscle proteolysis. The increased protein breakdown is stimulated by stress hormones, mainly cortisol. Along with glucagon, cortisol also stimulates gluconeogenesis and glycogenolysis in the liver. In skeletal muscle, cortisol inhibits the translocation of glucose transporters to the cell membrane, thereby decreasing glucose uptake. Concomitantly, binding of cortisol to glucocorticoid receptors in skeletal muscle cells initiates a cascade that results in the up-regulation of genes related to the ubiquitin–proteasomal system (22). The increased levels of circulating free fatty acids and amino acids resulting from these catabolic reactions further stimulates glucagon secretion, which continues to steadily rise as hours turn to days without food (19). The increase in gluconeogenesis in the liver and the decreased utilization of hepatic glucose by peripheral tissues ensure that the brain is still being supplied with glucose, its preferred fuel source.

After about 2 days of fasting, we enter the starvation state, which is a metabolic state fully adapted to nutrient scarcity. Although starvation is not pertinent to this review, several important metabolic adaptations occur in this state, mainly driven by the need to preserve essential protein. Notably, circulating ketone bodies rapidly increase because of accelerated lipolysis, increased hepatic fatty acid oxidation, and accumulation of acetyl-CoA. Once ketone bodies become readily available, skeletal and cardiac muscle and the brain start using these substrates as primary energy sources. Importantly, ketone body production can be stimulated independently of fasting-induced starvation by carbohydrate restriction (i.e. ketogenic diets), chronic caloric restriction, and some hypoglycemic oral medications (SGLT-2 inhibitors). The main processes and effectors involved in the transition from fed to fasting states are summarized in Table 1. The time spent in each of these states varies depending on one's physical activity level and basal metabolic rate as well as the nutrient composition and caloric density of the last meal.

**Table 1.** Main processes and metabolic effectors involved in the fed to fasting state transition and the effect of aging on these parameters.

Metabolic state	Fed	Post-absorptive	Fasting
Time since meal	0–3 h	3–18 h	18–48 h
Glucose source	Exogenous (diet)	Glycogenolysis, gluconeogenesis	Gluconeogenesis
Main brain source of energy	Glucose	Glucose	Glucose
Main systemic signals	Insulin	Glucagon	Glucagon, cortisol
Main cellular sensors	mTOR	AMPK, sirtuins	AMPK, sirtuins
Effect of aging	↓ Insulin sensitivity ↓ Glucose tolerance ↓ Preabsorptive insulin response	↑ Glucagon sensitivity? ↓ Ghrelin	↓ Ghrelin ↓ Growth hormone ↑ Cortisol

The events described above summarize the complex and interconnected processes that are in place to regulate energy metabolism and maintain organismal homeostasis. Several aspects of these processes are altered or impaired as we age, leading to age-related energy metabolism dysregulation. Understanding the alterations that lead to this dysregulation and how TRE can interact with them is paramount to developing safe and effective regimens that can be applied in clinical practice.

### Age-related changes in energy metabolism

The age-related changes in energy metabolism described below are complex and incompletely understood. Importantly, these changes affect several other physiological pathways as well as one another. To add to this complexity, they also depend on one's health status and health trajectory over time.

#### Insulin response and glucose tolerance

Decreases in insulin response and glucose tolerance are one of the most investigated age-related changes in energy metabolism. These changes are associated with multiple factors that lead to decreases in insulin secretion and tissues’ sensitivity to insulin (23, 24). At the onset of the fed state (i.e. when we start eating a meal), the initial sensory stimulation (smell, taste, texture, appearance, temperature) kickstarts the cephalic phase of digestion, where signals from the oral cavity reach the central nervous system, which then starts preparing the body for nutrient absorption by stimulating several digestive processes such as exocrine (e.g. saliva, gastric juices) and endocrine (e.g. insulin, leptin, pancreatic polypeptide, cholecystokinin) secretions, and gastric motility (25, 26). In other words, this preabsorptive mechanism aids the transition from catabolic to anabolic metabolism. Despite well-documented age-related changes in physiological processes that play a role in cephalic responses, their outcomes in age-related energy metabolism dysregulation remain unexplored. For instance, normal aging is associated with loss of taste and smell, impaired taste bud function and structure, and decreased saliva production (27–30). These changes may dampen cephalic responses to food consumption, impairing initial insulin response and post-prandial glucose handling (31, 32).

Insulin secretion itself is impaired during aging. Beta cells secrete insulin in two different pulse patterns: rapid and ultradian pulses. Rapid pulses have low amplitude and high frequency (every 8–15 min), and their main effect is inhibition of hepatic glucose production. Ultradian pulses have high amplitude and low frequency (every 60–140 min), and their main effect is stimulation of glucose uptake by peripheral tissues (33). In two complementary studies on age-related changes in insulin secretion, Meneilly et al. (34, 35) reported disruptions in both pulse patterns during fasting and hyperglycemic conditions. During hyperglycemia and fasting, rapid insulin pulses in older adults had lower amplitude than those of young adults. During hyperglycemia, ultradian pulses in older adults had lower frequency, amplitude, and regularity than those in young adults; during fasting, ultradian pulses had lower frequency in older than in young adults (34, 35). Taken together, these changes may contribute to the decrease in blood glucose control observed during aging.

Regarding peripheral glucose utilization, Rowe et al. (36) utilized a hyperinsulinemic–euglycemic clamp and increasing insulin doses to construct an insulin–glucose dose–response curve. Despite no changes in maximal glucose uptake, compared with young adults, older adults needed a higher dose of insulin to stimulate the disposal of the same amount of glucose (i.e. the dose–response curve was shifted to the right). Importantly, these observations were significant even when the results were corrected for lean body mass (36), indicating that these age-related changes in peripheral insulin sensitivity are independent of changes in body composition. Nevertheless, increases in fat mass and decreases in skeletal muscle mass observed during aging (37) also play a major role in age-related insulin resistance (38). While changes in insulin secretion and response during aging have been extensively studied, age-related changes in glucagon secretion and response have been less interrogated. Few studies conducted between 1970 and 1990s have reported no age-related changes in basal glucagon levels and alpha cell sensitivity to amino acids (39–41). Interestingly, Simonson and DeFronzo (41) observed an increase in glucagon-stimulated hepatic glucose production in older compared with young adults, which may contribute to elevated fasting blood glucose levels in this population. However, further investigations are required to elucidate this possible age-related change in glucagon sensitivity and its consequence to glucose control.

#### Systemic signaling changes

Aging is also associated with changes in several other hormones that participate in energy metabolism regulation. In the previous subsection, we highlighted the cephalic phase of digestion as the initial preabsorptive insulin response mediator. Now, as food moves through the digestive tract, other mechanisms are activated to further stimulate this initial insulin response. Incretins—namely glucagon-like peptide-1 (GLP-1) and insulinotropic polypeptide (GIP)—are released from enteroendocrine cells in response to the presence of nutrients in the small and large intestine (42). These hormones stimulate insulin secretion as part of the gut-endocrine pancreas axis. Studies exploring the effect of age on incretin responses have reported that GIP and GLP-1 responses are either unaltered or exacerbated with aging (40, 43–45); at the same time, these studies reported that the effect of incretins on beta cell insulin secretion is attenuated, indicating an age-related dysfunction in the gut-endocrine pancreas axis. Combined with a possible dampened cephalic insulin response, these observations suggest that the preabsorptive insulin response, which plays an important role in metabolic flexibility, is impaired during aging.

Other important hormones in metabolism regulation are ghrelin, growth hormone, and cortisol. Ghrelin is secreted by P/D1 epsilon cells located in the stomach and duodenum in response to sympathetic nervous system activation by stimuli such as food deprivation and stress (46). In the absence of exogenous fuel (i.e. during fasting), ghrelin acts to maintain blood glucose levels within a normal range (47). In the presence of exogenous fuel (i.e. when we consume a meal), the effects of elevated preprandial ghrelin levels persist to aid in dietary lipid utilization, increasing lipid storage in adipose and hepatic tissues while increasing fat oxidation in muscle tissue (48). Further, ghrelin (particularly the acylated isoform) is involved in growth hormone secretion due to its role as a growth hormone secretagogue receptor agonist (49). In turn, growth hormone is secreted from somatotropic cells located in the pituitary gland in response to nutritional and glycemic status, sleep, and stress. Like ghrelin, the effects of growth hormone depend on the nutritional context. During the fed state, growth hormone indirectly stimulates protein synthesis via the growth hormone/insulin-like growth factor 1 (IGF-1) axis. During the fasting state, growth hormone stimulates lipolysis and decreases peripheral insulin sensitivity, which aids in blood glucose homeostasis. Cortisol is secreted from zona fasciculata of the adrenal gland in response to adrenocorticotropic hormone stimulation and stress. Its main metabolic function is to maintain adequate availability of glucose for the brain, which it accomplishes by up-regulating gluconeogenesis in the liver and down-regulating glucose uptake and utilization in fat and muscle (50).

Importantly, aging is associated with a decrease in circulating ghrelin and growth hormone levels (51, 52) and with an increase in cortisol levels (53). In an informative study that monitored changes in plasma ghrelin, growth hormone, and cortisol over 24 h, Nass et al. (52) showed that mean acylated ghrelin levels were significantly lower in older compared with young adults and that the peak in acylated ghrelin concentration observed during sleep was absent in older adults. These changes in acylated ghrelin profile were accompanied by a decrease in mean growth hormone levels in older adults. This study (52) also demonstrated that the acylated ghrelin–growth hormone association is weakened during aging, indicating that growth hormone secretory pulse amplification is more dependent on other mechanisms (e.g. growth hormone releasing hormone) in older adults as opposed to what is observed in young adults (54). Finally, the study reported that mean cortisol levels were significantly higher in older compared with young adults due to pronounced increases in overnight cortisol levels (52). Taken together, the effect of aging on ghrelin, growth hormone, and cortisol leads to impaired utilization of dietary lipids, decreased lipolysis, and increased muscle proteolysis. Phenotypically, these changes can manifest as loss of muscle mass and increase in ectopic fat accumulation, which have detrimental functional and physiological consequences.

#### Cellular energy sensing changes

Aging is associated with alterations in the activity of cellular energy sensors. These alterations result from upstream energy metabolism dysregulation (e.g. impaired glucose homeostasis) and/or other factors that affect their activity independent of their main metabolic signals. For instance, studies have reported an age-related increase in mTOR activity in skeletal muscle tissue of aged mice and older adults (55, 56). Importantly, these studies reported no differences in the overall activity of the IGF-1/Akt pathway (56) and basal muscle protein synthesis rate (55) in older compared with young adults, indicating a dampened protein synthesis response. Hyperactivation of mTOR and its downstream target, S6K1, also contributes to peripheral insulin resistance (57), further impairing metabolic regulation.

On the catabolic spectrum of metabolism, AMPK activity and NAD^+^ bioavailability are also altered with aging. Since the landmark publication of Reznick et al. in 2007 (58) reporting on the age-associated decrease in AMPK activation in rats, following studies have contributed compelling evidence on AMPK activation insensitivity during aging (59–61). Li et al. (62) reported lower phosphorylation of AMPK and acetyl-CoA carboxylase in skeletal muscle of older men, which were not improved upon exercise training. As a critical node for metabolism regulation and stress response, defects in AMPK activity have propagating detrimental effects on the activity of downstream targets such as forkhead box transcription factors (mainly FoxO), nicotinamide phosphoribosyltransferase (NAMPT; involved in NAD^+^ biosynthesis), and sirtuins. Age-related NAD^+^ depletion also has systemic effects due to its role as a coenzyme in redox reactions and as an essential cofactor to many enzymes. This depletion is mainly attributed to an increase in NAD^+^-consuming pathways and a decrease in the activity of NAMPT, a rate-limiting enzyme involved in the NAD^+^ salvage pathway (63–65).

## Energy metabolism dysregulation and cerebrovascular aging

The human brain consumes one-fifth to one-quarter of one's total daily energy requirement, making it the most metabolically active organ in the human body (66). This high metabolic activity has two major consequences: (ⅰ) the brain milieu is exposed to elevated levels of metabolic waste and by-products and (ⅱ) bereft of sufficient intracellular energy storage, the major organ needs a constant supply of glucose and oxygen. In this context, cerebral blood vessels have specialized functions to meet the brain's needs. Structurally, cerebral vessels form the blood–brain barrier (BBB), an important physical barrier that separates the peripheral blood from the delicate neural environment. Besides keeping harmful molecules out, cerebral vessels facilitate the entry of selected molecules into the neural environment due to a specialized transcriptional profile of transporters genes (67). Further, cerebral vessels participate in the regulation of cerebral blood flow (CBF) via several mechanisms (68, 69). One of these mechanisms is termed neurovascular coupling (NVC), which couples neuronal activity to vasodilation using a complex intercellular signaling cascade involving neurons, astrocytes, smooth muscle cells, and endothelial cells (70). Therefore, the brain vasculature plays an indispensable role in the maintenance of brain health and function, and age-related metabolic dysfunction affecting the brain vasculature contributes to the development and aggravation of brain pathologies, including cognitive impairment and dementia.

Age-related changes in the cerebrovasculature have been extensively reviewed elsewhere (71, 72). Briefly, aging is associated with widespread decline in cerebrovascular health driven by arterial inflammation, increased arterial stiffness, increased BBB permeability, microvascular rarefaction, and decreased cerebrovascular reactivity (72). This decline deprives the neural environment of nutrients, oxygen, and waste clearance. Phenotypically, these changes can lead to cognitive decline and an increased susceptibility to cerebrovascular and neurological diseases. In this section, we will explore how the age-related changes in energy metabolism discussed in the previous section may precede and/or exacerbate cerebrovascular aging.

Age-related decreases in insulin sensitivity and glucose tolerance lead to impaired insulin signaling and hyperglycemia, which are potent inducers of oxidative stress and inflammation. Endothelial cells are especially vulnerable to hyperglycemia because they express glucose transporters constitutively and at high levels (GLUT1), unlike other tissues such as skeletal muscle and adipose, both of which need insulin to induce the expression of glucose transporters (GLUT4). In this way, high glucose levels in circulation cause a proportional increase in glucose levels inside endothelial cells. Hyperglycemia, in turn, leads to excess reactive oxygen species (ROS) via several mechanisms. First, high intracellular glucose levels decrease the activity of glucose-6-phosphate dehydrogenase (G6PD), an enzyme involved in the rate-limiting step of the pentose phosphate pathway, which yields ribose-5-phosphate and NADPH. The endogenous antioxidant system depends on adequate levels of NADPH to regenerate reduced forms (glutathione and thioredoxin) and maintain high activity levels (catalase and superoxide dismutase) of antioxidant enzymes (73, 74). Thus, hyperglycemia—via impaired G6PD function—deals a devastating blow to the antioxidant system, tipping the scales in favor of oxidative stress. Excess ROS increase damage to susceptible intracellular components such as proteins and DNA. Importantly, DNA strand breaks induce the activation of poly-ADP-ribose polymerase 1 (PARP1). Besides repairing DNA, PARP1 also modifies GAPDH, an enzyme involved in glycolysis, decreasing its activity. A decrease in GAPDH activity results in slowed glycolytic flux and accumulation of upstream substrates. These substrates are then diverted to alternative pathways, namely the polyol, hexosamine, glycation, and protein kinase C pathways (75–77). These mechanisms exacerbate ROS production, adding more weight to the already unbalanced cellular redox state.

These initial insults to the redox homeostasis of endothelial cells contribute to the initiation and aggravation of age-related cerebrovascular dysfunction. First, excessive ROS activates proinflammatory factors such as nuclear factor kappa B, contributing directly and reciprocally to arterial inflammation (including atherosclerosis, a chronic inflammatory condition) (78, 79). In turn, arterial inflammation begets arterial stiffening, which reduces arterial compliance and increases flow pulsatility, contributing to the development of hypertension and disruption of cerebral hemodynamics (80–83). Further, advanced glycation end products formed via the alternative glycation pathway aggravate arterial stiffness by crosslinking collagen fibers and increasing their content in the arterial wall (84, 85).

The structural integrity of the BBB is maintained by tight junctions, which comprise transmembrane and cytoplasmic proteins associated with the actin cytoskeleton. Intracellular ROS disrupts signaling pathways involved in tight junction maintenance and activates metalloproteinases, leading to actin rearrangement and transmembrane proteins redistribution and degradation (86, 87). In this way, hyperglycemia-induced oxidative stress increases BBB permeability, compromising the integrity and function of this important physical barrier. Furthermore, hyperglycemia-induced oxidative stress contributes to impaired angiogenesis. With the decreased glycolytic flux described previously, the production of lactate as a glycolysis product is also decreased. Lactate acts as a proangiogenic signaling molecule via several complementary pathways involving Akt, IL-8, and vascular endothelial growth factors, among others (88). Thus, changes in the rate of glycolysis that reduce lactate availability contribute to microvascular rarefaction, increasing the brain's susceptibility to hypoxic insults.

Another consequence of PARP1 activation is NAD^+^ depletion since this is a major NAD^+^-consuming pathway. Decreased NAD^+^ availability results in a decrease in the activity of other NAD^+^ -dependent enzymes such as sirtuins. Sirtuins are involved in the cellular stress response and are particularly important for adequate mitochondrial function. In endothelial cells, mitochondria play a major role in redox signaling and calcium homeostasis (89, 90). These factors contribute to nitric oxide (NO) bioavailability, a ubiquitous vasoactive gasotransmitter critical to homeostatic CBF regulation and cerebrovascular reactivity (91). Therefore, dysfunctions in mitochondrial function may initiate a cascade of events that culminate in abnormal endothelial nitric oxide synthase (eNOS) activity and impaired NO signaling. Excessive ROS production by dysfunctional mitochondria is a major driver of eNOS uncoupling, a condition in which the enzymology of eNOS changes from NO to superoxide radical production (92). Further, superoxide reacts with NO to form peroxynitrite (ONOO^−^), a potent oxidant that can exacerbate both eNOS and mitochondrial dysfunction (93). In this way, endothelial mitochondria and eNOS become intertwined in a vicious cycle of increasing damage that contributes to decreased cerebrovascular reactivity and inadequate tissue perfusion.

**Fig. 1.**
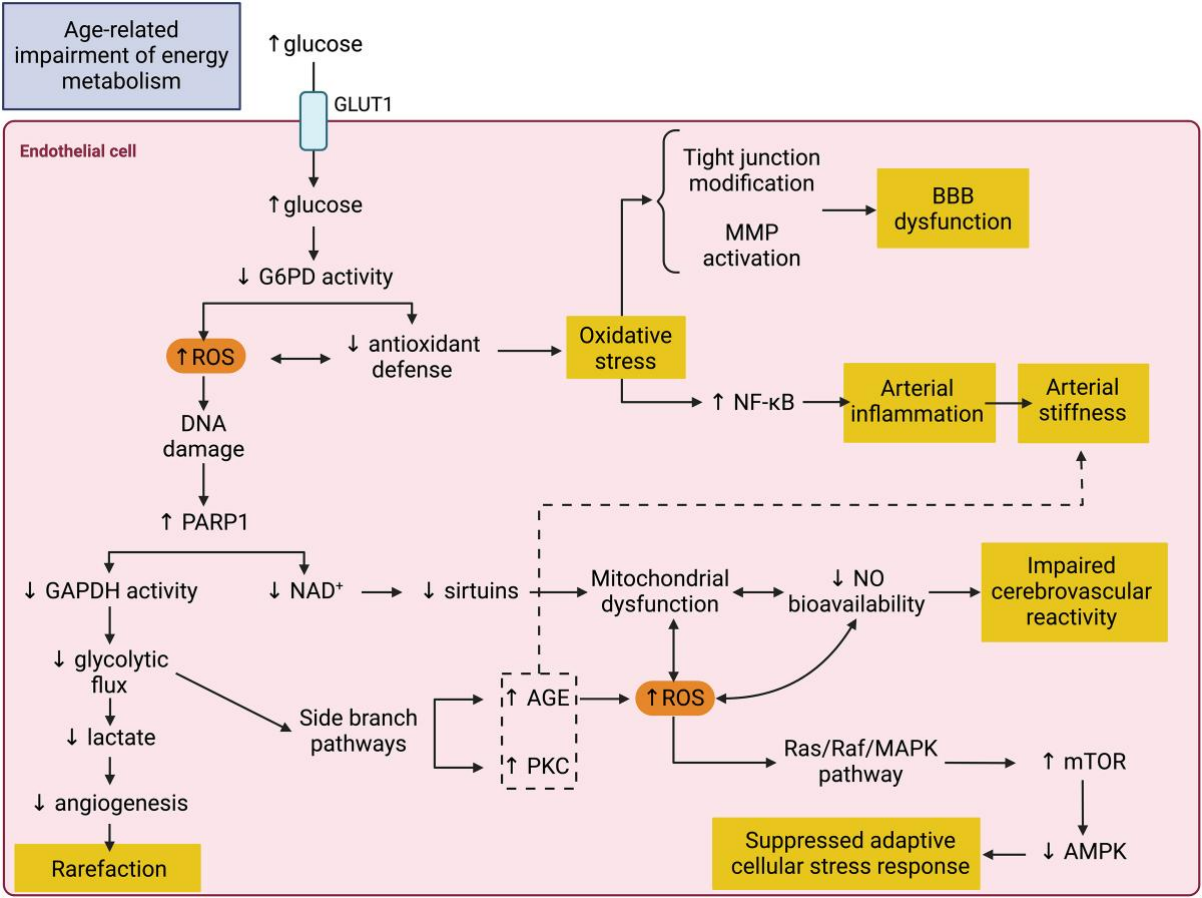
Schematic representation of how age-related energy metabolism dysregulation can initiate and propagate signaling cascades associated with cerebrovascular aging in endothelial cells. AGE, advanced glycation end products; GAPDH, glyceraldehyde 3-phosphate dehydrogenase; MMP, matrix metalloproteinase; mTOR, mammalian target of rapamycin; NAD, nicotinamide adenine dinucleotide; NF-κB, nuclear factor kappa B. Created with BioRender.com.

Another way in which hyperglycemia-induced oxidative stress can suppress the cellular adaptive stress response involves mTOR. ROS can activate the Ras/Raf/MAPK pathway, which in turn can activate mTOR independent of the PI3K mechanism (94). This may explain why mTOR seems overactive in older adults despite no alterations in the PI3K/Akt pathway, albeit this observation was made in skeletal muscle (56). Although mTOR is important for normal cellular function, excessive and sustained growth signaling has well-established detrimental effects on aging-related processes. Chief among these detrimental effects is the suppression of AMPK-mediated adaptive cellular stress responses and the cellular waste disposal system, leading to the accumulation of dysfunctional organelles and proteins. AMPK also participates in angiogenesis (88); thus, mTOR overactivation that suppresses AMPK signaling plays a role in microvascular rarefaction. Finally, the age-associated decrease in circulating acylated ghrelin and misalignment with growth hormone secretion (52) may contribute to the IGF-1 deficiency observed in aging. Importantly, IGF-1 insufficiency is associated with cerebrovascular aging, especially microvascular rarefaction and atherosclerosis, possibly due to its role in maintaining redox homeostasis in endothelial cells (95, 96). Taken together, the evidence presented in this section and summarized in Fig. 1 illustrates how age-related energy metabolism dysfunction and hyperglycemia sit atop a cascade of interconnected pathways that contribute directly and indirectly to cerebrovascular aging.

## Connecting the dots: how can TRE support cerebrovascular health during aging?

To explore how TRE can support healthy cerebrovascular aging, we summarized available studies on TRE in older adults. We conducted a search on PubMed and Scopus utilizing the search string “(intermittent fasting OR TRE) AND (aging OR older adults)” to identify these studies. This search returned 77 articles, which were then screened using the following inclusion criteria: (ⅰ) observational or interventional human studies, (ⅱ) participants aged 50 years or older, (ⅲ) TRE regimen, (ⅳ) published in English, and (ⅴ) published in the last 5 years. Of 77 articles initially retrieved, 12 met the inclusion criteria and are summarized here. One additional article was identified through reference list screening. Table 2 presents the characteristics of interventional studies that utilized TRE in older adults. Most studies have focused on anthropometric and cardiometabolic outcomes, and they have consistently reported improvements in these. At the same time, as evidenced by significant decreases in body weight reported by several studies (4, 98, 101, 103), caloric restriction is a common confounder; thus, whether the metabolic changes reported in these studies were due to TRE or caloric restriction remains undetermined.

**Table 2.** Characteristics of interventional human studies on TRE in older adults published in the last 5 years.

First author	Year	*N* (% male)	TRE regimen (h of fasting/h of free eating)	Inclusion age range (years)	Duration (weeks)	Main outcomes	Main findings
Kortas JA (97)	2024	24 (NR)	14:10	>60	12	Changes in ferritin, HbA1c, and glucose during a TRE plus exercise intervention	Decrease in ferritin (*P* = 0.01), HbA1c (*P* < 0.01), and glucose (*P* = 0.05) levels
Domaszewski P (98)	2023	116 (49%)	16:8	65–74	6	Changes in body weight, BMI, fat mass, skeletal muscle mass, waist circumference, and visceral fat mass	Decrease in body weight (*P* = 0.03) in both sexes; decrease in visceral fat mass (*P* < 0.001) and waist circumference (*P* < 0.015) in men
Boujelbane MA (99)	2022	58 (45%)	14:10^a^	>60	4	Changes in cognitive performance and sleep parameters during TRE based on physical activity level	Cognitive performance improvement (executive function, attention, inhibition, memory; all *P* < 0.04) in the physically active group only. Both groups had decreased sleep quality
Saini SK (100)	2022	9 (33%)	16:8	>64	4	Changes in circulating miRNA expression	Down-regulation of miRNAs associated with cell growth pathways (*P* < 0.05)
Domaszewski P (101)	2022	46 (100%)	16:8	65–74	6	Changes in body weight, BMI, fat mass, skeletal muscle mass, waist circumference, and visceral fat mass	Decrease in body weight (*P* < 0.001), BMI (*P* = 0.001), fat mass (*P* < 0.001), waist circumference (*P* < 0.001), and visceral fat mass (*P* < 0.001)
Andriessen C (102)	2022	14 (50%)	10:14	50–75	3	Hepatic glycogen, insulin sensitivity, and glucose homeostasis	Increased nonoxidative glucose disposal rate (*P* = 0.04), increased time spent in normoglycemia (*P* = 0.01), decreased fasting glucose (*P* = 0.03), and decreased 24-h glucose levels (*P* < 0.01)
Martens CR (3)	2020	24 (45%)	16:8	55–79	6	Safety, tolerability, and feasibility parameters; cardiovascular health parameters	Good safety, tolerability, and feasibility results. Increase in cardiovascular fitness (*P* < 0.05).
Domaszewski P (103)	2020	45 (0%)	16:8	>60	4	Changes in body weight, BMI, fat mass, skeletal muscle mass, waist circumference, and visceral fat mass	Decrease in body weight (*P* = 0.001), BMI (*P* = 0.001), and fat mass (*P* = 0.001)
Anton SD (4)	2019	10 (40%)	16:8	>64	4	Changes in body weight, waist circumference, cognitive and physical function, health-related quality of life, and adverse events	Good safety and adherence results. Decrease in body weight (*P* = 0.009) and BMI (*P* = 0.013)

SD, standard deviation; NR, not reported.

^a^Ramadan diurnal intermittent fasting.

One study authored by Martens et al. (3) assessed diet during the intervention and was designed to minimize caloric restriction. Despite no change in weight, they still observed improvements in glucose tolerance measured by oral glucose tolerance test, although they did report no changes in macrovascular endothelial function and insulin homeostasis parameters (3). In another study that recruited men with prediabetes between the ages of 35 and 70 years (mean age: 56 ± 9 years) (5), participants who adhered to an early TRE regimen (i.e. with the eating window early in the day) without weight loss displayed decreased fasting and post-prandial insulin levels, increased insulin sensitivity, and decreased lipid oxidative stress (measured by 8-isoprostane levels). However, they did not observe any changes in other oxidative and inflammatory markers, glucose homeostasis parameters, or arterial stiffness measures. Taken together, these studies indicate that TRE can be effective in improving glucose homeostasis in healthy older adults and insulin sensitivity in patients with prediabetes independently of caloric restriction. Importantly, considering the impact of glucose dysregulation as an upstream inducer of pathophysiological changes associated with cerebrovascular aging (see previous section), these results merit further investigation of TRE as a possible dietary intervention for supporting healthy cerebrovascular aging. Nonetheless, the short duration of these studies (5–6 weeks) may have been insufficient to observe changes in other relevant parameters.

Two additional studies on TRE employed a design that attempted to minimize concurrent weight loss (102, 104). Zhao et al. (104) recruited men with obesity between 40 and 70 years of age (mean age: 63 ± 4 years) and reported decreases in fasting glucose and glycated hemoglobin levels. Andriessen et al. (102) recruited adults with Type 2 diabetes between the ages of 50 and 75 years (mean age: 68 ± 5 years) and reported improvements in glucose control parameters. However, these two studies still reported unintended and significant weight loss, indicating that some degree of caloric restriction occurred during the intervention. These examples highlight that unintentional caloric restriction during TRE is common, which—besides acting as a confounder in clinical studies—may be undesirable in some older populations at increased risk of undernutrition, sarcopenia, and frailty.

Table 3 summarizes the characteristics of four observational studies that explored associations between eating time duration and health outcomes in larger population cohorts. These studies defined short eating time windows (<10 h) as adherence to a TRE regimen based on self-reporting of mealtimes. Interestingly, two studies on cognitive status reported opposite results: while Li et al. (11) found that TRE is associated with poorer cognitive function in a Chinese cohort, Currenti et al. (10) found that TRE is associated with better cognitive function in an Italian cohort. Although it is possible that the practice of TRE affects distinct populations differently, other factors should be considered as well. These studies did not make a distinction between voluntary and involuntary TRE, which is a source of confounding for observational studies. Voluntarily adhering to a TRE regimen is different than unintentionally eating for a shorter period of time. Restrictive eating patterns may instead reflect other prevalent aging issues such as depression and anorexia (107, 108). Further, cognitive impairment itself negatively affects dietary habits, often leading to poor oral intake, impaired hunger cues, and disinterest in food (109). In this way, these cross-sectional associations do not provide causative evidence of the effect of TRE on cognition. However, they do indicate that cognitive and mental status should be assessed prior to prescribing a TRE regimen to a patient or study participant. Taken together, the evidence summarized in this section shows the potential of TRE to improve energy metabolism and physiological outcomes in older adults; however, none of these studies investigated the effect of this dietary intervention on the cerebral vasculature.

**Table 3.** Characteristics of population-based observational human studies on TRE in older adults published in the last 5 years.

First author	Year	Number of participants	Population/cohort	Comparators	Main outcomes	Main findings
Li J (11)	2023	1,353	Community-dwelling Chinese older adults (>60 years old)	Eating time duration: >10 h vs <10 h	Cognitive domains based on C-MMSE scores	Lower overall C-MMSE scores, with lower orientation and attention/calculation domain scores, and higher prevalence of cognitive impairment (all *P* < 0.001) in those eating <10 h per day
Currenti W (10)	2021	916	Community-dwelling Italian older adults (>49 years old)/MEAL study cohort	Eating time duration: >10 h vs. <10 h	Cognitive status based on SPMSQ	Those eating <10 h a day were less likely to have cognitive impairment (OR 0.28 [CI 0.07–0.90])
Currenti W (105)	2021	1,572	Community-dwelling Italian older adults (>59 years old)	Eating time duration: >8 h vs. <8 h	Mental health parameters	Those eating <8 h a day and older than 70 years were less likely to present with mental distress (OR 0.14 [CI 0.03–0.65])
Estrada-DeLeón DB (106)	2021	1,226	Community-dwelling Spanish older adults (>63 years old)/Seniors-ENRICA-II cohort	Fasting duration: shortest (<10 h) vs. longest (>11 h) tertile	Lower extremity function assessed with SPPB	Longest fasting duration had a higher likelihood of lower extremity function impairment compared with shortest fasting duration (OR 2.70 [CI 1.80–4.04])

C-MMSE, Chinese Mini Mental State Examination; CI, confidence interval; OR, odds ratio; SPMSQ, Short Portable Mental Status Questionnaire; SPPB, Short Physical Performance Battery.

## Proof-of-concept findings on TRE and cerebrovascular health in older adults

To date, direct evidence of the effect of TRE on cerebrovascular parameters is lacking. The few preclinical studies on the topic have used surgical cerebral hypoperfusion or occlusion as a model of neurovascular diseases, thus limiting their interpretation in the context of normal aging. Rajeev et al. (110) reported that 4 months of 16-h daily fasting prior to bilateral common carotid artery stenosis attenuated cerebral microvessel leakage, BBB disruption, and tight junction damage in C57BL/6NTac mice compared with those fed ad libitum during the same period. Liu et al. (111) observed improvements in microvascular density, endothelial cell proliferation, and localized CBF after middle cerebral artery occlusion in Sprague Dawley rats that had undergone 3 months of 16-h daily fasting prior to surgery compared with their ad libitum counterparts.

**Fig. 2.**
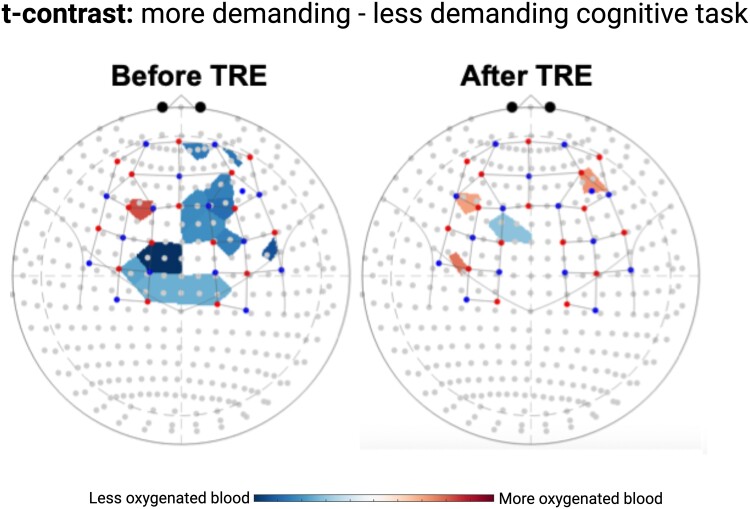
Effect of a 6-month 14:10 TRE regimen in older women on NVC responses assessed using functional near-infrared spectroscopy. The heatmap illustrates the distribution of oxygenated hemoglobin in cortical tissues during a working memory paradigm (N-back task), comparing responses before and after TRE intervention in six participants (mean age: 60.2 ± 3.9 years). Red and blue shaded areas indicate statistically significant increases and decreases, respectively, in NVC during the performance of more (2-back) vs. less challenging (1-back) tasks. More cognitively demanding tasks are expected to increase oxygenated hemoglobin concentrations at a greater magnitude compared with less cognitively demanding tasks.

As a proof-of-concept, our group has conducted a pilot study to determine the feasibility of a 6-month 14:10 TRE regimen in adults aged 55 years or older. The main outcome of this pilot study was change in NVC response from baseline to endpoint. As described previously, NVC is a primarily endothelium-dependent mechanism that matches CBF to neuronal activation to support brain function and reflects cerebrovascular health (112). This hemodynamic mechanism is associated with cognitive performance in young and older adults (113). NVC response was measured with functional near-infrared spectroscopy during a working memory paradigm (N-back task), which assesses changes in oxygenated and deoxygenated hemoglobin concentrations in cortical tissues upon neuronal activation as described elsewhere (114). More cognitively, demanding tasks are expected to increase oxygenated hemoglobin concentrations at a greater magnitude than less cognitively demanding tasks (115). In our limited dataset (*n* = 6, 100% female sex, mean age: 60.2 ± 3.9 years, compliance with regimen: 84.1%), we observed significant improvements in NVC responses in the prefrontal cortex after 6 months of TRE (Fig. 2). Based on self-reported dietary data (24-h dietary recalls) collected at multiple time points during the intervention period and total daily energy expenditure (TDEE) estimation, on average, participants met 99.3% (SD 19.5%; *n* = 5 [one participant was excluded due to implausible dietary data]) of their minimum TDEE for maintaining an isocaloric energy balance. The change in NVC response presented in Fig. 2 provides the first evidence that long-term adherence to a TRE regimen in human participants can affect the cerebrovasculature, aiding in the effective coupling of neuronal and vascular mechanisms involved in healthy brain function. However, the molecular and cellular mediators of this effect remain unclear, and larger studies must be conducted to confirm and expand on our preliminary data.

## Conclusion and future directions

TRE is a well-tolerated and promising dietary intervention for promoting and maintaining cerebrovascular health in older adults, mainly due to its beneficial effects on glucose homeostasis. Although stimulation of ketogenesis is also proposed as a mechanism responsible for the benefits associated with TRE, evidence for this (i.e. via increased circulating ketone bodies such as acetoacetate and beta-hydroxybutyrate) in the context of short-term TRE (14–16 h of fasting) is lacking. Of all studies identified in Table 2, only one (3) assessed acetoacetate and beta-hydroxybutyrate circulating levels before and after TRE and reported no changes. Thus, further studies designed for untangling the effects of fasting itself from the effects of caloric and/or carbohydrate restriction on ketogenesis in older adults are needed.

TRE—especially when accompanied by unintended caloric restriction—is not without its drawbacks, especially when applied to older adults. Inadequate energy intake in older adults can worsen aging-related bone density decline and skeletal muscle mass loss, thus increasing the risk of osteoporotic fractures and frailty (116, 117). In this way, we recommend that clinicians and researchers looking to apply TRE regimens in older adults be especially cognizant of these unique risks and evaluate (ⅰ) whether weight loss is advisable for their population of interest and (ⅱ) whether participants present with other conditions that can increase their risk of unintended weight loss such as malnutrition, depression, and functional limitations. Further studies on TRE in older adults must be better controlled for energy balance and of longer duration to extricate its independent effects from those of caloric restriction, especially when weight loss is not an outcome of interest (118).
